# An Observational Study on the Clinical Characteristics and Prognosis of Patients With Interstitial Lung Disease Secondary to Dermatomyositis and Antisynthetase Syndrome

**DOI:** 10.1155/2024/9679944

**Published:** 2024-09-26

**Authors:** Ling Lei, Zongbo Ma, Xuejia Ma, Dongmei Pan, Zhanrui Chen, Fang Qin, Fei Dong

**Affiliations:** Department of Rheumatology and Immunology The First Affiliated Hospital of Guangxi Medical University, Nanning, Guangxi Province 530021, China

**Keywords:** antisynthetase syndrome, dermatomyositis, idiopathic inflammatory myopathy, interstitial lung disease, prognosis

## Abstract

**Objective:** Identify the clinical characteristics and prognostic factors in patients with idiopathic inflammatory myopathy (IIM) combined with interstitial lung disease (ILD).

**Methods:** IIM-ILD patients who were hospitalized at Guangxi Medical University from January 2017 to December 2022 were retrospectively analyzed and classified as having dermatomyositis (DM)-ILD or -ILD. Clinical and laboratory results were analyzed.

**Results:** There were 39 males and 111 females, the mean age of disease onset was 50.4 ± 12.3 years, and the median disease duration was 3 months (range: 1–6). Ninety-seven patients had DM-ILD, and 53 had ASS-ILD. The DM-ILD group had 72% positivity for the anti-MDA5 antibody and 5.2% positivity for the anti-Mi-2 antibody; the ASS-ILD group had 67.9% positivity for the anti-Jo-1 antibody and 17% positivity for the anti-EJ antibody. Muscle symptoms, skin ulcers, rash, rapidly progressing interstitial lung disease (RP-ILD), and elevated levels of serum carcinoembryonic antigen were more common in DM-ILD patients (all *p* < 0.05). However, pericardial effusion and pleural effusion, elevated creatinine kinase, and elevated C-reactive protein were more common in ASS-ILD patients. After a median follow-up of 15.5 months, there were more deaths in the DM-ILD group (42.3% vs. 13.2%, *p* < 0.001). Multivariate Cox regression analysis showed that RP-ILD, dyspnea, and the usual interstitial pneumonia type of ILD had negative associations with overall survival (OS), and arthralgia had a positive association with OS (all *p* < 0.05).

**Conclusion:** DM-ILD patients were more prone to secondary RP-ILD and skin ulcers, had milder symptoms of myositis and less severe serositis, and had lower survival rates than the ASS-ILD patients. RP-ILD, dyspnea, and the usual interstitial pneumonia type of ILD had adverse effects on prognosis, but arthralgia was a protective factor.

**Summary**

• DM-ILD patients had higher prevalences of RP-ILD, skin ulcers, and elevated carcinoembryonic antigen and lower prevalences of pleural effusion and pericardial effusion.
• Relative to our ASS-ILD patients, our DM-ILD patients had a significantly higher overall mortality rate and a significantly lower 5-year cumulative survival rate.
• RP-ILD, dyspnea, and the usual interstitial pneumonia type of ILD had negative associations with overall survival (OS), and arthralgia had a positive association with OS.

## 1. Introduction

Idiopathic inflammatory myopathy (IIM) is an autoimmune disease whose major symptoms are muscle weakness and cutaneous inflammation. Patients with different subtypes of IIM vary in their clinical manifestations, therapeutic responses, and prognosis. Based on clinical manifestations, histopathology, and serology, most IIM cases are classified as dermatomyositis (DM) or antisynthetase syndrome (ASS); the less common subtypes include immune-mediated necrotizing myopathy (IMNM), inclusion body myositis, polymyositis (PM), and overlap myositis [1, 2]. Other than the skin and muscular system, interstitial lung disease (ILD) is the most common systemic involvement and the main cause of death in patients with IIM. ILD in these patients often manifests as cough, dyspnea, and hypoxemia, with audible wet rales in the lungs and chest imaging results showing ground-glass shadows, reticular shadows, honeycomb shadows, and solid shadows.

Recent studies reported that the incidence of ILD in IIM patients overall ranged from 21.9% to 74.8% and that the incidence differed for the different subtypes (DM: 73.6%, ASS: 75.0%, PM: 55.1%, and IMNM: 30%) [3, 4]. For patients with DM, those who are positive for anti–melanoma differentiation-associated gene 5 (MDA5) often present with rapidly progressive interstitial lung disease (RP-ILD), and these patients have poor prognosis. ILD is often more common in ASS patients, a class of IIM defined by positivity for the anti–aminoacyl tRNA synthetase (ARS) antibody, and severe ILD is also associated with poor prognosis in these patients. The Gottron sign, V-sign, periorbital purple edema spot, and other skin symptoms are common in DM-ILD patients; myasthenia, arthralgia/inflammation, and mechanic hands are common in ASS-ILD patients [1]. Patients with different subtypes of IIM differ in their clinical features, autoantibodies, developmental course, and response to treatment.

The purpose of the present study is to compare the clinical features, diagnosis, treatment, and prognosis of patients with DM-ILD and ASS-ILD and identify factors that affect the prognosis of these patients to improve understanding of this condition.

## 2. Materials and Methods

### 2.1. Study Subjects

The records of 150 consecutive patients with DM-ILD or ASS-ILD who were hospitalized in the Department of Rheumatology and Immunology of the First Affiliated Hospital of Guangxi Medical University from January 1, 2017, to December 30, 2022, were retrospectively examined. These patients were divided into a DM-ILD group and an ASS-ILD group.

#### 2.1.1. Inclusion Criteria

The inclusion criteria for both groups were age of at least 18 years, complete medical history and clinical data, and diagnosis of DM-ILD or ASS-ILD. DM was diagnosed according to the 1975 Bohan/Peter criteria [5] or the 2017 European League Against Rheumatism/American College of Rheumatology (EULAR/ACR) criteria for the classification of adult and juvenile forms of inflammatory myopathies and their major subtypes [2]. ASS was diagnosed as proposed by Li et al. [6]. ILD was diagnosed by the presence of at least one of the following two findings: (i) chest X-ray suggestive of pulmonary fibrosis, with or without pulmonary function tests suggestive of restrictive ventilatory dysfunction, or (ii) high-resolution computed tomography (HRCT) of the chest with one or more of four manifestations (grid shadow, ground-glass shadow, honeycomb sign, and tractive bronchiectasis). RP-ILD [7] was defined by the progressive deterioration of ILD within 3 months, with worsening of exertional shortness of breath and acute progressive respiratory failure, progressive chest HRCT manifestations, and a decrease of 1.33 kPa or more in the arterial blood partial pressure of oxygen.

#### 2.1.2. Exclusion Criteria

The exclusion criteria for both groups were as follows: age below 18 years; co-occurrence of a connective tissue disease, such as systemic lupus erythematosus, rheumatoid arthritis, systemic vasculitis, Sjögren's syndrome, and systemic sclerosis; ILD with a clearly defined etiology (pneumoconiosis, drugs, allergic pneumonia, radiation pneumonitis, etc.); presence of a severe cardiovascular, cerebrovascular, or hepatic disease or renal insufficiency; presence of an uncontrollable lung infection; and absence of complete medical history and clinical data.

### 2.2. Clinical Indicators

#### 2.2.1. General Information and Clinical Presentation

The following patient characteristics were recorded at presentation: general information (age at disease onset, gender, duration of disease, and smoking status); clinical characteristics, including initial respiratory symptoms (cough, chest tightness, and dyspnea); skin and muscle symptoms (inflammation, aches, and weakness); fever; arthralgia; subcutaneous/mediastinal emphysema; dysphagia; Raynaud's phenomenon; cutaneous ulcers; Velcro bronchial sounds on auscultation; specific type of rash (Gottron sign, Gottron's papules, periungual telangiectasia with cuticular hemorrhage and dystrophy, mechanic's hand, heliotrope rash, and poikilo-DM); gastrointestinal involvement; cardiac involvement; and pulmonary arterial hypertension (PAH).

Gastrointestinal involvement was defined by the presence of acid reflux, distending pain in the upper abdomen, diarrhea, malabsorption, and other symptoms. Cardiac involvement was defined by symptoms such as chest tightness, chest pain, palpitations, and shortness of breath; arrhythmia or conduction block on an electrocardiogram; echocardiography suggestive of valvular anomalies, pericarditis, or lowered ejection fraction; and systolic pressure greater than 40 mmHg in the pulmonary arteries.

#### 2.2.2. Laboratory Tests

The routine blood analysis included measurements of cells, metabolic markers, cancer markers, immunological indicators, and blood gas analysis. Myositis autoantibodies were detected using a microarray chip (TestLine Clinical Diagnostics s.r.o.) combined with ELISA (3450-2, Shanghai Feisheng Biotechnology Co., Ltd). Electrocardiogram, gastroenteroscopy, echocardiography, and lung function tests were also performed.

The chest HRCT results were read by an experienced rheumatologist and radiologist and evaluated for the most compatible imaging classification according to the 2013 American Thoracic Society/European Respiratory Society (ATS/ERS) diagnostic criteria for idiopathic ILD [8]. The patients were classified into five groups based on these results: (i) nonspecific interstitial pneumonia (NSIP), defined by predominantly basal distribution in bilateral lungs with ground-glass and grid shadows, which may be accompanied by tractional bronchiectasis, with or without a mild honeycomb sign; (ii) usual interstitial pneumonia (UIP), defined by predominantly subpleural distribution of the honeycomb sign in the lower regions of both lungs, with or without grid shadows and tractional bronchiectasis; (iii) organizing pneumonia (OP), defined by peripheral, bronchial vascular bundle or flaky distribution of a dominant solid shadow, with or without air bronchial signs, and often accompanied by ground-glass shadow; (iv) acute interstitial pneumonia (AIP), defined by predominantly diffuse or bilateral patchy ground-glass shadows [9]; and (v) unclassified interstitial pneumonia (U-ILD), defined by atypical HRCT findings that cannot be identified as a specific imaging type, or a mixture of two or more imaging types that cannot be classified. When there was disagreement regarding the classification, the two clinicians discussed the decision until reaching an agreement.

The Siemens Force dual-source CT (US GE256 slice spiral CT) was used for imaging. All patients were in a supine position, with their arms raised and their bodies in the center of the examination table. The horizontal line was aligned with the midline of the armpit, and the scanning area covered the entire chest. The scanning parameters were as follows: tube voltage 90–120 kV, tube current 60–120 mA, reconstruction layer thickness 0.625–1.0 mm, and matrix 512 × 512. The lung algorithm was used for reconstruction. The clinical window was set as the lung window, with a width of 1500 HU and a level of −500 HU.

### 2.3. Treatments, Follow-Up, and Prognosis

Treatment efficacy, pulmonary infection, and survival times were determined during follow-up, as indicated in the outpatient records, rehospitalization records, or telephone calls. The survival rates at 1, 3, and 5 years were calculated. Survival rate calculations excluded patients who died from malignant tumors or infections.

### 2.4. Statistical Methods

SPSS Version 26.0 was used for statistical analysis. For comparisons of the two groups, measurement data were compared using the independent samples *t*-test or the rank-sum test, and count data were compared using the *χ*^2^ test. The Kaplan–Meier method was used to analyze survival times, and the log-rank test was used to compare the survival of the two groups. Cox regression analysis was used to identify factors associated with overall survival (OS). A *p* value below 0.05 was considered statistically significant.

## 3. Results

### 3.1. Characteristics of the DM-ILD and ASS-ILD Groups

#### 3.1.1. General Characteristics and Autoantibodies

We analyzed the records of 150 patients with IIM-ILD, 97 with DM-ILD and 53 with ASS-ILD (Table 1). Overall, there were 39 males and 111 females, the mean age of disease onset was 50.4 years (± 12.3), and the median duration of disease was 3 months (range: 1–6).

We analyzed the presence of different autoantibodies in the two groups (data not shown). Analysis of the DM-ILD group indicated positivity for the anti-MDA5 antibody in 70 patients (72.2%), for the anti-NXP2 antibody in two patients (2.1%), for the anti-Mi-2 antibody in five patients (5.2%), for the anti-TIF-1*γ* antibody in four patients (4.1%), and for the anti-SAE antibody in one patient (1.0%). Analysis of the ASS-ILD group indicated positivity for the anti-ARS antibody in all 53 patients (100%), for the anti-Jo-1 antibody in 37 patients (67.9%), for the anti-EJ antibody in nine patients (17%), for the anti-PL-12 antibody in five patients (9.4%), for the anti-PL-7 antibody in four patients (7.5%), and for the anti-SRP antibody in two patients (3.8%). Positivity for the anti-Ro52 antibody was significantly greater in the ASS-ILD group (81.1% vs. 55.7%, *p* = 0.003).

#### 3.1.2. Clinical and Laboratory Characteristics

We also compared the clinical characteristics of the two groups (Table 1). Overall, fever (50%) and arthralgia (69.3%) were the most common symptoms, and 33 patients (22%) presented with respiratory symptoms as the first symptom. Thirty-one patients (20.7%) had RP-ILD, and the prevalence was significantly greater in the DM-ILD group (30.9% vs. 1.9%, *p* < 0.05). Type I respiratory failure had a prevalence of 17.5% in the DM-ILD group and 7.5% in the ASS-ILD group (*p* = 0.092). The prevalences of skin/muscle symptoms, skin ulcers, and specific rash were greater in the DM-ILD group (all *p* < 0.05), but the prevalences of pleural effusion and pericardial effusion were greater in the ASS-ILD group (both *p* < 0.05). The incidence of pulmonary infection in the DM-ILD and ASS-ILD patients was 40.2% and 49.1%, respectively (with no statistical difference).

Blood samples were available for all 150 patients (Table 2). The ASS-ILD group had greater levels of white blood cells (WBCs), platelets (PLTs), creatinine kinase (CK), C-reactive protein (CRP), and immunoglobulin M (IgM) and a greater percentage of patients with an elevated cancer antigen 125 (CA125; all *p* < 0.05). The DM-ILD group had a significantly greater percentage of patients with an elevated level of carcinoembryonic antigen (CEA, *p* < 0.05).

#### 3.1.3. Pulmonary Function Tests and Chest Imaging

Data from pulmonary function tests were available for 39 patients (69.6%) in the DM-ILD group and 17 patients (30.4%) in the ASS-ILD group (data not shown). Overall, 40 patients (71.4%) had diffusion impairment, and 27 patients (48.2%) had restrictive ventilation. The DM-ILD and ASS-ILD groups had no significant differences in forced expiratory volume in 1 s (FEV1), forced vital capacity (FVC), FEV1/FVC, carbon monoxide diffusing capacity (DLCO), vital capacity (VC), residual volume (RV), and total lung capacity (TLC). The chest HRCT results showed that NSIP was the most common manifestation in both groups and that the prevalence of AIP was greater in the DM-ILD group (18.6% vs. 3.8%, *p* = 0.005, Table 3).

#### 3.1.4. Treatments

Patients were treated with glucocorticoids (prednisone, methylprednisolone, etc.), immunosuppressants, tofacitib, rituximab, and/or antipulmonary fibrosis drugs (nintedanib and pirfenidone) (Table 4).

### 3.2. Survival Analysis and Prognostic Factors

#### 3.2.1. Survival Analysis

The follow-up time ranged from 1 to 69 months, and the median was 15.5 months. Analysis of the treatment regimens (data not shown) demonstrated that all 150 patients received glucocorticoids and 140 patients (93.3%) received combined immunosuppressant therapy. Five patients (3.3%) received CD20 monoclonal antibody, and two of them died. Seventeen patients (11.3%) received tofacitib, and six of them died. Two patients (2.1%) in the DM-ILD group developed malignant tumors; the patient with lymphoma died, and the other patient with renal cancer survived as of the last follow-up.

**Figure 1:**
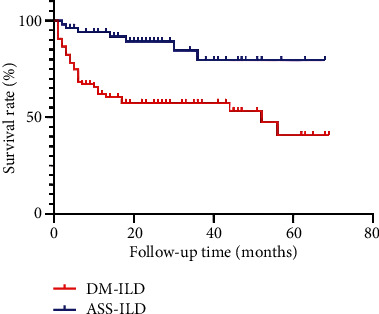
Kaplan–Meier survival analysis of the DM-ILD group and ASS-ILD group.

A significantly higher percentage of patients died in the DM-ILD group (42.3% vs. 13.2%, *p* < 0.001, Figure 1). The 1-, 3-, and 5-year survival rates were 63.9%, 60.8%, and 57.7% in the DM-ILD group and 94.3%, 88.7%, and 86.8% in the ASS-ILD group, respectively. A log-rank test showed that survival time was significantly longer in the ASS-ILD group (*χ*^2^ = 14.275, *p* < 0.001).

#### 3.2.2. Prognostic Factors

We performed a Cox regression analysis to identify factors associated with OS (Table 5). The results of the univariate analysis showed that smoking, dyspnea, mediastinal emphysema, gastrointestinal involvement, comorbid RP-ILD, Type I respiratory failure, pulmonary infection, elevated CEA, and anti-MDA5 antibody positivity were negatively associated with OS; disease duration of more than 6 months, myalgia, arthralgia, anti-Jo-1 antibody positivity, and ANA positivity were positively associated with OS. In addition, patients with the UIP and AIP subtypes of ILD had an increased risk of death compared to those with the NSIP subtype.

We then entered all factors that were significant in the univariate analysis into a multifactorial Cox regression model. The results showed that RP-ILD, dyspnea, and the UIP subtype of ILD had independent and negative associations with OS and that arthralgia had an independent and positive association with OS (all *p* < 0.05).

## 4. Discussion

ILDs are a group of diffuse lung diseases that primarily involve the interstitial and alveolar spaces of the lungs and can result in the loss of alveolar–capillary functional units. Patients with ILD typically present with progressive dyspnea, audible Velcro rales in the lungs, and hypoxemia, and the lung function test results often indicate restrictive ventilation dysfunction with reduced diffusion function [10], the most common and serious complications in our group of ASS and DM patients. Our study showed that fever and arthralgia were the most common symptoms in these patients, and respiratory symptoms, such as cough and dyspnea, were initial symptoms in 22% of our patients. Previous research found that ILD occurred in 51.9%–100% of patients with ASS [11, 12] and that there was a gradual increase over time in the number of ASS patients who initially presented with respiratory symptoms [13]. Compared with our ASS-ILD patients, our DM-ILD patients had a higher incidence of RP-ILD and skin ulcers. Several studies confirmed the poor prognosis of DM patients who presented with acute exacerbations of ILD and specific skin ulcers [14–16]. We also found that DM-ILD patients had a higher prevalence of elevated CEA, but a lower prevalence of elevated CA125, lower levels of CRP, CK, and CK-MB, and lower prevalences of pleural effusion and pericardial effusion. This suggests that DM-ILD myopathy was relatively mild in our patients and that the onset may be related to changes in tumor markers and less occurrence of serositis. Zuo et al. [17] found that serum CA153 levels were elevated in patients with anti-MDA5+DM secondary RP-ILD, which is also an independent risk factor for ASS-ILD in secondary RP-ILD; elevated serum CEA and CA125 levels are also independent risk factors for ASS-ILD secondary to RP-ILD [18].

Two of our DM-ILD patients developed malignant tumors, corresponding to an incidence rate of 2.1%. Previous studies showed that the incidence of tumors in patients with DM ranged from 20% to 25%, significantly higher than that in our study. Other research suggested that ILD may be negatively correlated with malignant tumors [19, 20], so this may explain our discrepant findings. Tumorigenesis is uncommon in patients with ASS-ILD, and a study by Pinal-Fernandez et al. [21] found no significant increase in mortality or cancer risk in patients with ASS compared to the general population in the United States. Taken together, these findings suggest that IIM patients with tumorigenesis predominantly have DM and that DM patients without ILD have a greater risk of cancer than DM patients with ILD.

Our chest HRCT results showed that NSIP was the most common CT manifestation in both groups and that the prevalence of AIP was greater in the DM-ILD group than in the ASS-ILD group. Our multivariate analysis showed that RP-ILD, dyspnea, and UIP manifestations were independent risk factors for poor prognosis. An IIM-ILD patient who develops RP-ILD has a poor prognosis, and the risk of death remains high even when high doses of corticosteroids and immunosuppressants are administered. Cobo-Ibáñez et al. [22] showed that the risk of death in patients with IIM was associated with the presence of RP-ILD, severe infections, and a sun rash within 3 months of illness, consistent with our results. UIP presents as interstitial reticular or honeycomb changes in both lungs, with progressive exacerbations, and predominantly fibrotic lesions. A recent study found that the incidence of UIP in ASS patients was higher in those who were positive for PL-7 autoantibodies (13.3%) than those with another autoantibody status; moreover, these PL-7+ patients had the least improvement in FVC after corticosteroid and immunosuppressive therapy, the poorest response to therapy overall, and the poorest prognosis [23]. For patients with early-stage ILD, monitoring by chest HRCT is needed for the timely detection of RP-ILD, AIP, and UIP, so that active interventions can be given promptly to improve prognosis.

Relative to our ASS-ILD patients, our DM-ILD patients had a significantly higher overall mortality rate (42.3% vs. 13.2%) and a significantly lower 5-year cumulative survival rate. Previous studies showed that the overall mortality rate of DM and PM patients was 14% [24], the 1-year survival rate of ASS patients was 75% [25], and the mortality rate of DM-ILD patients ranged from 53.0% to 68.5% [26, 27]. All of these results suggest that our DM-ILD group had more serious disease than our AAS-ILD group and that the presence of ILD was responsible for the increased mortality of DM patients.

We found that disease duration of more than 6 months, myalgia, arthralgia, anti-Jo-1 antibodies, and ANA positivity were associated with better prognosis in IIM patients and that arthralgia was independently and significantly associated with a better prognosis. Cobo-Ibáñez et al. [22] also showed that ANA positivity had a protective effect in female patients with IIM and that joint manifestations were associated with a reduced risk of worsening respiratory function, consistent with our findings. Mehta, Agarwal, and Gupta [25] found that arthralgia, rash, and ANA positivity were associated with a reduced risk of IIM and adverse outcomes in patients from India; in agreement, another study reported that ANA negativity was a predictor of poor prognosis in patients from Australia [28]. A study by Hozumi et al. [29] showed that ASS-ILD patients had a higher 10-year survival rate than DM-ILD patients and that anti-Jo-1 antibody-positive patients had a better prognosis than antibody-negative patients [30]. We also found that DM-ILD patients with disease duration greater than 6 months had a relatively good prognosis, and the positive rate of anti-Ro-52 antibody is higher than that of ASS-ILD patients. Consistent with previous studies by Chinese scholars, the RP-ILD incidence rate of patients with a positive anti-Ro52 antibody is higher [31–33], which is related to the acute exacerbation of IIM-ILD in the early stage of the disease [34]. This indicates that the acute exacerbation of DM-ILD disease in the early stage may be related to the positive rate of anti-Ro-52 antibody, which is the main reason for poor prognosis.

In conclusion, our comparison of clinical characteristics and prognosis of patients with secondary ILD caused by DM or ASS showed that 20.7% of patients had RP-ILD and 30.9% of DM-ILD patients developed RP-ILD. Patients with DM-ILD usually have relatively mild myopathy, and the onset of the disease may be related to different tumor markers, and they were less likely to present with serositis. Our DM-ILD group had a significantly worse OS rate than our ASS-ILD group. For our patients overall, the presence of RP-ILD, dyspnea, and manifestations of UIP were independent risk factors for poor prognosis, although patients with a disease duration greater than 6 months, ANA positivity, and arthralgia had a better prognosis.

## Figures and Tables

**Table 1:** General characteristics of the DM-ILD group and ASS-ILD group.

**Characteristic**	**Total (** **n** = 150**)**	**DM-ILD (** **n** = 97**)**	**ASS-ILD (** **n** = 53**)**	**χ** ^2^/**F**/**Z value**	**p** ** value**
Age of onset (years)	50.4 ± 12.3	49.4 ± 10.7	52.2 ± 14.8	−1.226	0.224
Gender					
Male	39 (26%)	27 (27.8%)	12 (22.6%)	0.480	0.488
Female	111 (74%)	70 (72.2%)	41 (77.4%)		
Disease duration (months)	3 (1, 6)	3 (1, 5)	3 (1.5, 12)	−1.931	0.053
Initial hospitalization (days)	13 (10, 18)	14 (10.5, 19)	12 (9, 17)	−2.113	0.035
Smoking	26 (17.3%)	17 (17.5%)	9 (17%)	0.007	0.933
Respiratory symptoms^a^	33 (22%)	20 (20.6%)	13 (24.5%)	0.305	0.581
Muscle symptoms	68 (45.3%)	53 (54.6%)	15 (28.3%)	9.593	0.002
Fever	75 (50%)	49 (50.5%)	26 (49.1%)	0.029	0.864
Arthralgia	104 (69.3%)	64 (66%)	40 (75.5%)	1.452	0.228
Dysphagia	22 (14.7%)	18 (18.6%)	4 (7.5%)	3.319	0.068
Mediastinal emphysema	7 (4.7%)	7 (7.2%)	0		0.052^∗∗^
Raynaud phenomenon	17 (11.3%)	12 (12.4%)	5 (9.4%)	0.294	0.588
Gastrointestinal involvement	8 (5.3%)	6 (6.2%)	2 (3.8%)	0.062	0.804
Cardiac involvement	12 (8%)	7 (7.2%)	5 (9.4%)	0.027	0.87
Skin ulcers	20 (13.3%)	20 (20.6%)	0	12.609	< 0.001
Velcro rales	48 (32%)	30 (30.9%)	18 (34%)	0.145	0.703
Specific rash	109 (72.7%)	92 (94.8%)	17 (32.1%)	67.988	< 0.001
RP-ILD	31 (20.7%)	30 (30.9%)	1 (1.9%)	17.630	< 0.001
Pericardial effusion	26 (17.3%)	10 (10.3%)	16 (30.2%)	9.453	0.002
Type I respiratory failure	21 (14%)	17 (17.5%)	4 (7.5%)	2.834	0.092
Lung infection	65 (43.3%)	39 (40.2%)	26 (49.1%)	1.093	0.296
Pleural effusion	29 (19.3%)	11 (11.3%)	18 (34%)	11.247	0.001
Pulmonary hypertension	25 (16.7%)	12 (12.4%)	13 (24.5%)	3.647	0.056

*Note:* Data are presented as mean ± SD, *n* (%), or median (range).

^a^Respiratory symptoms as these patients' first manifestation.

^∗∗^Fisher exact probability method.

**Table 2:** Laboratory indices of the DM-ILD group and ASS-ILD group.

**Index**	**Total (** **n** = 150**)**	**DM-ILD (** **n** = 97**)**	**ASS-ILD (** **n** = 53**)**	**t** ** or ** **Z** ** value**	**p** ** value**
WBC (×10^9^/L)	6.93 (4.815, 10.41)	6.12 (4.34, 8.245)	8.95 (6.87, 13.9)	−4.303	< 0.001
HGB (g/L)	116.3 (105.4, 125)	116 (104.3, 124.65)	116.3 (108.25, 125.45)	−0.374	0.709
PLT (×10^9^/L)	283.034 ± 118.3714	256.775 ± 101.42	331.092 ± 132.3606	−3.557	0.001
CK (U/L)	177.5 (68, 769.5)	104 (50, 338)	712 (163.5, 2436.5)	−5.255	< 0.001
LDH (U/L)	370.5 (300.75, 529.5)	362 (300.5, 456.5)	422 (308, 614)	−1.659	0.097
ALT (U/L)	39 (22, 89.75)	37 (22.5, 75.5)	48 (19.5, 135.5)	−0.788	0.43
AST (U/L)	55.5 (32, 117)	56 (35, 110.5)	47 (28.5, 145)	−0.617	0.537
ALB (g/L)	31.381 ± 5.3338	31.136 ± 4.9035	31.828 ± 6.0675	−0.759	0.449
GLO (g/L)	32.55 (28.9, 38.325)	32.3 (27.9, 37.4)	33.8 (29.6, 39.25)	−1.335	0.182
CRP (mg/L)	7.895 (3.475, 22.745)	6.17 (2.38, 17.105)	11.9 (5.3, 26.18)	−2.721	0.007
ESR (mm/h)	36 (17, 52)	36 (17, 51)	36 (16.5, 55)	−0.452	0.651
SF (ng/mL	1536.2 (435.28, 2206.153)	1441.8 (442.785, 2276.95)	1762.925 (389.86, 2145.62)	−0.538	0.591
IgA (g/L)	2.595 (1.955, 3.25)	2.62 (1.935, 3.17)	2.53 (1.97, 3.49)	−0.006	0.995
IgM (g/L)	1.645 (1.0525, 2.47)	1.45 (0.995, 2.21)	2.03 (1.3005, 2.715)	−2.206	0.027
Elevated CEA	38 (25.3%)	34 (35.1%)	4 (7.5%)	13.707	< 0.001
Elevated CA125	23 (15.3%)	7 (7.2%)	16 (30.2%)	13.932	< 0.001
Elevated CA153	35 (23.3%)	26 (26.8%)	9 (17%)	1.849	0.174
Elevated CA199	8 (5.3%)	5 (5.2%)	3 (5.7%)	0	1.000
ANA	91 (60.7%)	48 (49.5%)	43 (81.1%)	14.386	< 0.001
Anti-jo-1 antibody	46 (30.7%)	10 (10.3%)	36 (67.9%)	53.508	< 0.001
Anti-Ro52 antibody	97 (64.7%)	54 (55.7%)	43 (81.1%)	9.725	0.001

*Note:* Data are presented as median (range), *n* (%), or mean ± SD.

Abbreviations: ALB, albumin; ALT, alanine aminotransferase; AST, aspartate aminotransferase; CA125, cancer antigen 125; CA153, cancer antigen 153; CA199, cancer antigen 199; CEA, carcinoembryonic antigen; CK, creatine kinase; CK-MB, CK MB isoform; CRP, C-reactive protein; ESR, erythrocyte sedimentation rate; GLO, globulin; HGB, hemoglobin; IgA, immunoglobulin A; IgM, immunoglobulin M; LDH, lactate dehydrogenase; PLTs, platelets; SF, serum ferritin; WBCs, white blood cells.

**Table 3:** Chest CT manifestations of the DM-ILD group and ASS-ILD group.

**Group**	**n**	**NSIP**	**UIP**	**OP**	**AIP**	**U-ILD**
DM-ILD	97	52 (53.6%)	4 (4.1%)	19 (19.6%)	18 (18.6%)	4 (4.1%)
ASS-ILD	53	35 (66%)	7 (13.2%)	8 (15.1%)	2 (3.8%)	1 (1.9%)
*p* value	—	0.140	0.087	0.494	0.011	0.800

*Note:* Data are presented as *n* (%).

Abbreviations: AIP: acute interstitial pneumonia; NSIP: nonspecific interstitial pneumonia; OP: organizing pneumonia; U-ILD: unclassified interstitial pneumonia; UIP: usual interstitial pneumonia.

**Table 4:** Treatments received by patients in the DM-ILD group (*n* = 97) and the ASS-ILD group (*n* = 53). [Table-fn fn2]

**Treatment**	**Total**	**DM-ILD**	**ASS-ILD**	**χ** ^2^ ** value**	**p** ** value**
Corticosteroid	150 (100.0%)	97 (100.0%)	53 (100.0%)	/	/
MP pulse therapy	3 (2%)	2 (2.1%)	1 (1.1%)	/	0.715^∗^
Immunosuppressant	140 (93.3%)	91 (93.8%)	49 (92.5%)	0.102	0.497
Number of immunosuppressants^a^				13.173	0.003
0	8 (5.3%)	6 (6.2%)	2 (3.8%)		
1	60 (40%)	29 (29.9%)	31 (58.5%)		
2	65 (43.3%)	47 (48.5%)	58 (47.9%)		
3	17 (11.3%)	15 (15.5%)	2 (3.8%)		
Rituximab	5 (3.3%)	4 (4.1%)	1 (1.9%)	/	0.419^∗^
Tofacitinib	17 (11.3%)	14 (14.4%)	3 (5.7%)	2.625	0.085
Anti-PF drugs	52 (34.7%)	35 (36.1%)	17 (32.1%)	0.243	0.379
Gamma globulin	37 (24.7%)	32 (33%)	5 (9.4%)	10.234	0.001
Plasma exchange	4 (2.7%)	4 (4.1%)	0 (0.0)	/	0.171^∗^
Antibiotic	68 (45.3%)	43 (44.3%)	25 (47.2%)	0.112	0.435

*Note:* Data are presented as *n* (%).

Abbreviations: MP: methyprednisolone; PF: pulmonary fibrosis.

^a^Including mycophenolate mofetil, tacrolimus, cyclosporine, methotrexate, cyclophosphamide, leflunomide, azathioprine, hydroxychloroquine sulfate, thalidomide, and *Tripterygium wilfordii* glycosides.

^∗^Fisher's exact probability method.

**Table 5:** Cox regression analysis of factors associated with overall survival.

**Variable**	**Univariate**		**Multivariate**	
	**HR (95% CI)**	**p** ** value**	**HR (95% CI)**	**p** ** value**
Gender				
Female	0.667 (0.366, 1.216)	0.186		
Male	Ref.			
Age (years)				
> 55	1.166 (0.639, 2.127)	0.617		
≤ 55	Ref.			
Disease duration (months)				
> 6	0.312 (0.123, 0.789)	0.014	0.407 (0.141, 1.173)	0.096
≤ 6	Ref.		Ref.	
Myalgia	0.533 (0.287, 0.989)	0.046	1.189 (0.508, 2.784)	0.69
Arthralgia	0.398 (0.224, 0.708)	0.002	0.369 (0.184, 0.742)	0.005
Dysphagia	2.172 (1.129, 4.178)	0.02	1.234 (0.469, 3.249)	0.67
Mediastinal emphysema	6.031 (2.505, 14.519)	< 0.001	0.715 (0.234, 2.187)	0.556
Gastrointestinal involvement	2.798 (1.103, 7.101)	0.03	2.473 (0.614, 9.959)	0.203
Smoking	1.902 (1.005, 3.599)	0.048	1.439 (0.696, 2.976)	0.327
RP-ILD	7.324 (4.094, 13.1)	< 0.001	2.459 (1.082, 5.587)	0.032
Type I respiratory failure	9.735 (5.258, 18.025)	< 0.001	2.29 (0.927, 5.661)	0.073
Dyspnea	2.856 (1.479, 4.478)	0.002	2.491 (1.132, 5.48)	0.023
Lung infection	2.873 (1.595, 5.175)	< 0.001	1.655 (0.763, 3.591)	0.202
Elevated CEA	3.229 (1.813, 5.753)	< 0.001	1.735 (0.81, 3.715)	0.156
Type of ILD				
NSIP	Ref.		Ref.	
UIP	3.08 (1.132, 8.376)	0.028	3.12 (1.019, 9.549)	0.046
AIP	8.791 (4.359, 17.728)	< 0.001	2.417 (0.918, 6.359)	0.074
Anti-MDA5 antibody	3.13 (1.71, 5.728)	< 0.001	1.328 (0.556, 3.17)	0.523
Anti-Jo-1 antibody	0.285 (0.127, 0.637)	0.002	0.473 (0.175, 1.281)	0.141
ANA	0.542 (0.307, 0.955)	0.034	0.825 (0.425, 1.601)	0.569

Abbreviations: AIP: acute interstitial pneumonia; ANA: antinuclear antibodies; CEA: carcinoembryonic antigen; MDA5: melanoma differentiation-associated gene 5; NSIP: nonspecific interstitial pneumonia; UIP: usual interstitial pneumonia.

## Data Availability

The data that support the findings of this study are available on request from the corresponding author. The data are not publicly available due to privacy or ethical restrictions.
